# Iron regulates myeloma cell/macrophage interaction and drives resistance to bortezomib

**DOI:** 10.1016/j.redox.2020.101611

**Published:** 2020-06-24

**Authors:** Giuseppina Camiolo, Alessandro Barbato, Cesarina Giallongo, Nunzio Vicario, Alessandra Romano, Nunziatina L. Parrinello, Rosalba Parenti, Joaquín Cantón Sandoval, Diana García-Moreno, Giacomo Lazzarino, Roberto Avola, Giuseppe A. Palumbo, Victoriano Mulero, Giovanni Li Volti, Daniele Tibullo, Francesco Di Raimondo

**Affiliations:** a Section of Biochemistry, Department of Biomedical and Biotechnological Sciences, University of Catania, 95123, Catania, Italy; b Section of Hematology, Department of Medical and Surgical Specialties, A.O.U. Policlinico-OVE, University of Catania, 95125, Catania, Italy; c Section of Hematology, Department of Medical, Surgical Sciences and Advanced Technologies “G.F. Ingrassia”, A.O.U. Policlinico-OVE, University of Catania, 95125, Catania, Italy; d Section of Physiology, Department of Biomedical and Biotechnological Sciences, University of Catania, 95123, Catania, Italy; e Division of Hematology, A.O.U. Policlinico-OVE, University of Catania, 95122, Catania, Italy; f Departamento de Biología Celular e Histología, Facultad de Biología, Universidad de MurciaIMIB-Arrixaca, Centro de Investigación Biomédica en Red de Enfermedades Raras (CIBERER), Murcia, 30100, Spain; g UniCamillus - Saint Camillus International University of Health Sciences, Via di Sant’Alessandro 8, 00131, Rome, Italy

**Keywords:** Iron, Multiple myeloma, Zebrafish, Monocyte

## Abstract

Iron plays a major role in multiple processes involved in cell homeostasis such as metabolism, respiration and DNA synthesis. Cancer cells exhibit pronounced iron retention as compared to healthy counterpart. This phenomenon also occurs in multiple myeloma (MM), a hematological malignancy characterized by terminally differentiated plasma cells (PCs), in which serum ferritin levels have been reported as a negative prognostic marker. The aim of current study is to evaluate the potential role of iron metabolism in promoting drug resistance in myeloma cancer cells with particular regard to the interactions between PCs and tumor-associated macrophages (TAMs) as a source of iron. Our data showed that myeloma cell lines are able to intake and accumulate iron and thus, increasing their scavenger antioxidant-related genes and mitochondrial mass. We further demonstrated that PCs pre-treated with ferric ammonium citrate (FAC) decreased bortezomib (BTZ)-induced apoptosis *in vitro* and successfully engrafted in zebrafish larvae treated with BTZ. Treating human macrophages with FAC, we observed a switch toward a M2-like phenotype associated with an increased expression of anti-inflammatory markers such as ARG1, suggesting the establishment of an iron-mediated immune suppressive tumor microenvironment favouring myeloma growth. Using *mfap4:tomato mutant* zebrafish larvae, we further confirmed the increase of PCs-monocytes interactions after FAC treatment which favour BTZ-resistance. Taken together our data support the hypothesis that targeting iron trafficking in myeloma microenvironment may represent a promising strategy to counteract a tumor-supporting milieu and drug resistance.

## Highlights

• Myeloma cell lines uptake iron and increase oxidative-redox related genes.
• Iron increases mitochondrial biogenesis and fitness in myeloma cell lines.
• Iron promotes bortezomib resistance in myeloma cell lines.
• Zebrafish xenografts of myeloma cell lines exposed to iron exhibit bortezomib resistance.
• Iron exposition promotes monocytes-plasma cells interactions and immunomodulation.

## Graphical abstract

**Figure:**
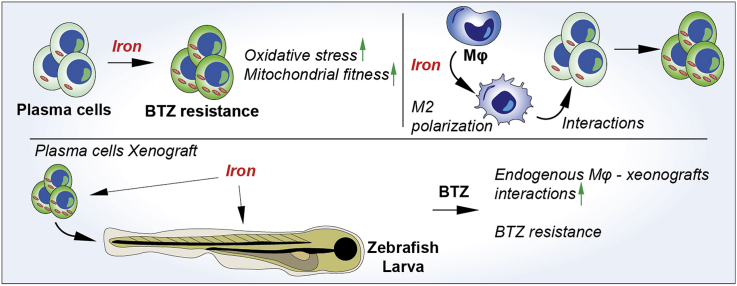

## 1 Introduction

Multiple myeloma (MM) is a hematological malignancy characterized by terminally differentiated plasma cells (PCs), immunosuppression and end-organ damage, including osteolytic lesions, renal failure, anemia and hypercalcemia [1]. MM progression is tightly dependent on bone marrow microenvironment which supports and regulates tumor proliferation, survival and migration of PCs. Despite the therapeutic progress achieved with the introduction of the proteasome inhibitors (PI), immunomodulators and monoclonal antibodies, MM remains a non-curable disease due to intrinsic or acquired drug resistance [2]. Bortezomib (BTZ) is a first-in-class selective PI, which has been proven to be effective in the treatment of MM. However, nearly all MM patients eventually relapse and approximately one third of them develops resistance to BTZ [3]. Therefore, additional studies are warranted in order to develop more effective therapies targeting myeloma cells and their microenvironment, which plays an important role in tumor progression and drug resistance.

Iron is essential for many biological functions including electron transport, DNA synthesis, detoxification and erythropoiesis, all contributing to metabolism, cell growth, and proliferation [4]. Moreover, it is involved in free-radical-generating reactions, including Fenton reaction, in which ferrous iron (Fe^2+^) donates an electron to hydrogen peroxide to produce the hydroxyl radical and other highly reactive oxygen species (ROS) [5]. Although excess of iron can induce toxic oxidative damage ensuing cell death and induce DNA damage leading to tumor transformation and growth [6]. Tumor cells regulate intracellular iron metabolism to favour iron accumulation by increasing iron uptake and storage, and, at the same time, decreasing iron export [7]. It seems that cancer cells retain most elements of the general iron metabolism pathway, although for many cancer types this has not been rigorously or explicitly studied. However, cancer cells differ from their non-malignant counterparts in the levels or activity of many of the proteins that are involved in iron metabolism. In many cases, the net result of these cancer-specific alterations is an increase in intra cellular iron levels that fuels the activity of iron-dependent proteins and enables enhanced proliferation. In some cases, the downstream consequences of changes in iron-regulated proteins remain unclear. In order to increase iron retention tumor cells express higher levels of iron import proteins, such as transferrin receptor (TFR1) and divalent metal transporter 1 (DMT1) [[8], [9], [10], [11], [12]]. To this regard, in leukemia patients there exists a relationship between dysregulation of iron metabolism and the occurrence and progression of the disease [13,14]. On account of their high requirement for iron to maintain their rapid proliferation, leukemia cells are more susceptible to iron depletion than normal cells [15]. Similarly, MM cells also exhibit altered iron metabolism allowing increased iron intracellular levels leading to cell proliferation and drug resistance [[16], [17], [18]]. A recent study found that ferroportin (FPN1), the only iron exporter in mammalian cells, is decreased in myeloma PCs leading to an increase in the labile iron pool (LIP) [19].

Furthermore, several studies highlighted the importance of iron-restricted erythropoiesis and anemia of chronic diseases triggered by myeloma-associated inflammation [20,21]. Most of the iron used to sustain erythropoiesis is released by macrophages that recycle iron into the bloodstream derived from senescent erythrocytes, through FPN1 [22]. Macrophages can acquire a range of different phenotypes and functions based on the environmental stimuli. In particular, tumor-associated macrophages (TAM) exhibit an alternative activated and suppressive (M2-like) phenotype and are likely guided by apoptotic cancer cells to release iron, lipocalin 2 and ferritin into the tumor microenvironment [23,24]. PCs myeloma affect macrophage behaviour shifting their phenotype toward a M2-like profile which support MM cells growth and protect them from chemotherapy-induced apoptosis [25]. To this regard, Bordini and colleagues previously demonstrated that iron excess in myeloma PCs promoted cell death and potentiated BTZ effect in a murine model of MM [26]. By contrast, deferasirox (DFO), an oral iron chelator used to treat iron overload in clinical practice, inhibits MM cell growth both *in vivo* and *in vitro* [27]. Therefore, the aim of the present work is to elucidate the significance of iron metabolism in MM cells, its effect on response to BTZ and to unravel the molecular mechanisms underlying PCs-macrophage interactions.

## 2 Material and methods

### 2.1 Cell culture and treatments

Human myeloma cell lines (U266, OPM2, NCI–H929) and U937 cell line were cultured in RPMI 1640 medium supplemented with 10% FBS and 1% penicillin/streptomycin at 37 °C and 5% CO_2_. Primary peripheral blood CD14^+^ monocytes were obtained by healthy donor buffy coat after separation by Ficoll-Hypaque gradient and cultured for 3 days in RPMI-1640 medium supplemented with 10% FBS and 1% Penicillin/streptomycin in presence of 10 nM Phorbol 12-myristate 13-acetate (PMA) at 37 °C and 5% CO_2_ [28].

Based on the previous literature data [29], 15 nM BTZ (Takeda, Rome, Italy) was used in all experiments. Used dose of ferric ammonium citrate (FAC) was 100 μM or 400 μM [[30], [31], [32]].

### 2.2 Apoptosis assay

Evaluation of apoptosis was performed by flow cytometry. Samples (5 × 10^5^ cells) were washed and resuspended in 100 μL of phosphate-buffered saline (PBS). 1 μL of Annexin V-FITC solution and 5 μl of Propidium Iodide (Beckmam Coulter, made in France) were added to cell suspension and mixed gently. Cells were incubated for 15 min in the dark. Finally, 400 μl of 1X binding buffer was added and cell preparation was analyzed by flow cytometry (MACSQuant Analyzer 10, Miltenyi Biotec).

### 2.3 Intracellular LIP estimation

To quantify LIP, 0.5 × 10^6^ cells were collected and washed with PBS. Then cells were incubated with 0,5 μM calcein acetoxymethyl ester (CA-AM) (Sigma- Aldrich) for 15 min at 37 °C. After cell washing, samples were incubated with a high-affinity chelator, 100 μM deferiprone (DF) (Sigma- Aldrich), at 37 °C for 1 h. Cells were washed 3 times in phosphate-buffered saline (PBS) at 1500 rpm for 5 min and then analyzed by flow cytometry (MACSQuant Analyzer 10, Miltenyi Biotec). The difference in the MFI before and after treatment with DF was used to calculate the amount of LIP (ΔF = MFI_CA-AM/DF_-MFI_CA-AM_).

### 2.4 Real-time RT-PCR for gene expression analysis

For each experiment, total RNA was extracted from cells using Trizol reagent and quantified using a UV spectrophotometer (NANODROP 1000, Thermofisher), as previously described [33]. One microgram of total RNA (in 20 μL reaction volume) was reverse-transcribed in cDNA using reverse-transcriptase (Applied Biosystem) and oligo-dT primers in a standard reaction. The quantitative real-time polymerase chain reaction (RT-PCR) of the resultant cDNA was performed using Sybr Green PCR Master Mix (ThermoFisher Scientific) and 7900HT Fast Real-Time PCR System (Thermo Fisher) [34,35]. Expression of the following human genes was evaluated: HMOX1 (FW: AAGACTGCGTTCCTGCTCAAC, RW: AAAGCCCTACAGCAACTGTCG); DMT1(FW: TGCATTCTGCCTTAGTCAAGTC, RW: ACAAAGAGTGCAATGCAGGA); FPN1 (FW: CATGTACCATGGATGGGTTCT, RW: CAATATTTGCAATAGTGATGATCAGG); ND4 (FW: ACAAGCTCCATCTGCCTACGACAA, RW: TTATGAGAATGACTGCGCCGGTGA); CYTB (FW: TCCTCCCGTGAGCGCGGTGA, RW: TTATGAGAATGACTGCGCCGGTGA); GLUT-S-TRANSFERASE (FW: CTGGGCTTCGAGATCCTGTG, RW: GGCAGACAAACTTCCACTGTC); TFAM (FW: GGTCTGGAGCAGAGCTGTGC, RW: TGGACAACTTGCCAAGACAGAT); SOD (FW: TGGTTTGCGTCGTAGTCTCC; RW: CCAAGTCTCCAACATGCCTCT); GST (Fw: CTGGGCTTCGAGATCCTGTG; Rw: GGCAGACAAACTTCCACTGTC); B2M (Fw: AGCAGCATCATGGAGGTTTG; Rw: AGCCCTCCTAGAGCTACCTG); GAPDH (Fw: AATGGGCAGCCGTTAGGAAA; Rw: GCCCAATACGACCAAATCAGAG). Gene expression analysis of pro-inflammatory and anti-inflammatory cytokines IL-6, CCL2, TNFα, TGFB1 and ARG1 was performed using GoTaq Master mix (Promega) according to manufacturer's recommended protocol. Each reaction was run in triplicate. For each sample, the relative expression level of the mRNA of interest was determined by comparison with the control housekeeping genes B2M and GAPDH using the 2^^−ΔΔCt^ method.

### 2.5 Immunofluorescence

For immunofluorescence, paraformaldehyde-fixed cells samples were permeabilized in 0.1% Triton X100 in PBS and incubated with blocking solution (10% normal goat serum, NGS, in 0.1% Triton X100 in PBS) for 1 h at room temperature [[36], [37], [38]]. Samples were incubated overnight at 4 °C with the following primary antibodies diluted in PBS Rabbit anti-NOS2 (Santa Cruz, Cat#sc-7271; 1:100), mouse anti-ARG1 (Santa Cruz, Cat#sc-20150; 1:100). The following day, after washing, samples were incubated for 1 h at room temperature with the appropriate fluorescence goat secondary antibodies: anti-rabbit 546 (Invitrogen, Cat# A11010, RRID: AB_143156, 1:1000) and anti-chicken 488 (Abcam, Cat# ab150169, RRID: AB_2636803, 1:1000). Nuclei were counterstained with 4’,6-diamidino-2-phenylindole (Dapi, 1:1000, Cat# D1306, Invitrogen) for 5 min at room temperature. Slides were mounted with fluorescent mounting medium Permafluor (ThermoScientific) and digital images were acquired using a Leica DM IRB (Leica Microsystems, Buccinasco, Milano, Italy) fluorescence microscope or the Leica TCS SP8 confocal microscope.

### 2.6 Flow cytometry

For apoptosis evaluation, 5 × 10^5^ cells were washed and resuspended in 100 μL of PBS. 1 μL of Annexin V-FITC solution and 5 μl of Propidium Iodide (Beckmam Coulter, made in France) were added to cell suspension and mixed gently. Cells were incubated for 15 min in the dark. Finally, 400 μl of 1X binding buffer was added and cell preparation was analyzed by flow cytometry (MACSQuant Analyzer 10, Miltenyi Biotec).

To determine the intracellular ROS levels, cells were stained with using the 2′,7′-Dichlorofluorescein (Sigma-Aldrich) and fluorescence intensity was measured according to the fluorescence detection conditions of FITC.

A membrane potential probe, the 3,3′-Diethyloxacarbocyanine Iodide (DiOC2(3)), was used to evaluate the mitochondrial membrane potential. Cells were incubated with 10 μM DiOC2(3) (Thermo Fisher Scientific, Milan, Italy) for 30 min at 37 °C, washed twice, resuspended in PBS and analyzed by flow cytometry through the detection of the green fluorescence intensity of DiOC2(3).

In order to measure changes in the mitochondrial mass, cells were reacted with 200 nM MitoTracker Red CMXRos probe (Thermo Fisher Scientific) for 30 min at 37 °C, according to the manufacturer's instructions. After being washed twice, labeled mitochondria were analyzed by flow cytometry.

To evaluate CD206, CD86, CD163, HLA-DR expression in U937 cells and primary human monocytes, cells were washed and resuspended in 100 μl of PBS. 10 μl of anti-HLA-DR-PC5 (clone Immu-357; Beckman Coulter), CD206-PE (clone 3.29B1.10; Beckman Coulter), CD86-PE (clone BU63; Biolegend) and CD163-PerCP (clone GHI/61; Biolegend) were added to each tube. Cells were incubated for 15 min at room temperature, protected from light. After centrifugation, cells were washed in 1 ml of PBS and analyzed using flow cytometer.

### 2.7 Myeloma xenograft model

Zebrafish (*Danio rerio* H.) larvae were obtained from the Zebrafish International Resource Center and mated, staged, raised and processed as described [39]. The line tg(*mfap4:Tomato)*^*xt12*^ [40] in Casper background has been previously described [41]. Zebrafish fertilized eggs were obtained from natural spawning of transgenic fish held at the facilities following standard husbandry practices. Animals were maintained for 12 h in light/dark cycle at 28 °C. The experiments performed comply with the Guidelines of the European Union Council (Directive 2010/63/EU) and the Spanish RD 53/2013. Experiments and procedures were performed as approved by the Bioethical Committees of the University of Murcia (approval numbers #75/2014, #216/2014 and 395/2017).

Zebrafish larvae 2 dpf were anesthetized by a solution of 0.16 mg/ml buffered tricaine in embryo medium (Sigma-Aldrich) followed by myeloma cells xenotransplantation, using a microinjector system (Narishige). Before the injection in the Cuvier duct, 2 × 10^6^ MM cells were pre-stained with Vybrant DiO cell-labeling solution (Thermofisher). Larvae were imaged using epifluorescence Lumar V12 stereomicroscope and the number of myeloma cells homed in the CHT were manually counted.

To evaluate macrophages interaction with myeloma cells, *mfap4:tomato transgenic* larvae were treated for 24 h with 100 μM FAC and subsequently injected with Vybriant DiO labeled MM cells.

### 2.8 Larvae manipulation for inflammation assay and macrophage polarization visualization

To evaluate the role of iron in guiding macrophage polarization, *Tg(mpeg1:mCherry;tnfa:eGFP)* double transgenic larvae, in which tnfa^+^ macrophages (M1) are eGFP and mCherry double-positive [42], were used. Transgenic larvae (30 for each group) were treated with 100 μM FAC and/or 100 μM Deferoxamine (DFO) in a 6 wells plate for 24 h. Caudal fin amputation was performed on 3 dpf larvae as described previously [43]. The caudal fin was transected with a sterile scalpel, posterior to muscle and notochord under anesthesia with 0.016% Tricaine (ethyl 3-aminobenzoate, SigmaAldrich, France) in zebrafish water. Macrophages expressing TNFα (M1) are shown as yellow puncta due to the merged mCherry-eGFP fluorescence, while macrophages not expressing TNFα (M2) are shown as red puncta (mCherry^+^eGFP^−^).

### 2.9 Sample preparation and chromatographic conditions of the HPLC analysis of metabolites

All ultrapure standards, used for the evaluation of cellular metabolic profile, tetrabutylammonium hydroxide and potassium di-hydrogen phosphate (KH2PO4) suitable for all buffer preparations, were purchased by Sigma-Aldrich (St. Louis, MO, USA) and diluted in Ultrapure water (18.3 MΩ cm) (Milli-Q Synthesis A10, Millipore, Burlington, MA, USA). HPLC-grade methanol, far-UV acetonitrile and chloroform were supplied by J. T. Baker Inc. (Phillipsburgh, NJ, USA).

Metabolic analysis was performed after deproteinization of cell samples (3 × 106 cells) according to a protocol suitable to obtain protein-free extracts for further HPLC analysis of acid labile and easily oxidizable compounds [18]. Cells were washed twice with PBS at pH 7.4 and collected by centrifugation at 1860×*g* for 5 min at 4 °C. Cell pellets were deproteinized with the addition of 1 ml of ice-cold, nitrogen-saturated, 10 mM KH2PO4 in CH3CN, pH 7.4 (1:3, v/v). After vigorous mixing for 60 s, samples were centrifuged at 20690 g for 10 min at 4 °C. The organic solvent was removed from the deproteinized supernatants using two washings with 5 ml of chloroform. The upper aqueous phase obtained by centrifugation at the same conditions, was then used for the HPLC analysis of low molecular weight metabolites. Simultaneous separation of 50 low molecular weight metabolites related to energy metabolism, oxidative/nitrosative stress, antioxidants, and including high energy phosphates (ATP, ADP, AMP, GTP, GDP, GMP, UTP, UDP, UMP, CTP, CDP, CMP, IMP), oxidized and reduced nicotinic coenzymes (NAD+, NADH, NADP+, NADPH), were carried out using a Hypersil C-18, 250 × 4.6 mm, 5 μm particle size column, provided with its own guard column (Thermo Fisher Scientific, Rodano, Milan, Italy), following slight modifications of previously established ion pairing HPLC methods [19,20]. The HPLC apparatus was a SpectraSYSTEM P4000 pump (Thermo Fisher Scientific) interfaced to a highly-sensitive UV6000LP diode array detector (Thermo Fisher Scientific), equipped with a 5 cm light path flow cell and set up between 200 and 300 nm wavelength. Assignments and calculations of the aforementioned compounds in cell extracts, were performed by comparing retention times, absorption spectra, and area of the peaks (calculated at 260 nm wavelength for all compounds but GSH, nitrite and nitrate that were calculated at 206 nm wavelength) of chromatographic runs of mixtures containing known concentrations of ultrapure standards.

### 2.10 Statistical analysis

Data are shown and expressed in the main text as mean value ± standard error of the mean (SEM) as absolute values or fold change (FC) over control. Data were tested for normality using Shapiro-Wilk normality test and subsequently assessed for homogeneity of variance. Data that does not meet the criteria of Shapiro-Wilk normality test were analyzed using Mann-Whitney *t*-test for comparison of n = 2 groups. Comparisons of n > 2 groups were performed using ANOVA and Kruskal-Wallis multiple comparisons test. All data with normal frequency distribution were analyzed by two-tailed unpaired Student's t-test for comparison of n = 2 groups, unless otherwise stated. Comparisons of n > 2 groups were performed using ANOVA and Sidak's multiple comparisons test, unless otherwise stated. For all statistical tests, p values < 0.05 were considered statistically significant.

## 3 Results

### 3.1 Myeloma cell lines are able to internalize iron

In order to elucidate the effects of iron in myeloma PCs, we exposed human myeloma cell lines (HMCLs) to 400 μM ferric ammonium citrate (FAC). In our preliminary set of experiment and being 24 h the maximum exposure timing, we first excluded that the chosen dose had no cytotoxic effects in HMCLs by evaluating the percentage of apoptotic cells following FAC treatment (Supplementary Fig. 1). Next, we evaluated mRNA levels of iron trafficking-related genes DMT-1 (divalent metals transporter 1) and FPN1 (ferroportin 1) observing a significant upregulation after 6 h FAC exposure both of DMT-1 (fold change (FC) over controls: 1.54 ± 0.05, 2.94 ± 0.06 and 1.54 ± 0.09 respectively in U266, OPM2 and NCI–H929 cell lines; Fig. 1A) and FPN1 (FC over controls: 1.44 ± 0.06 and 4.1 ± 0.04 in U266 and OPM2 cells, respectively; Fig. 1B) as compared to untreated control cultures. NCI–H929 and U266 cells preserved significantly higher FPN1 mRNA levels also at 24 h post FAC treatment (FC over controls: 2.5 ± 0.09 and 1.95 ± 0.05 respectively in U266 and NCI–H929 cells; Fig. 1B). To confirm the ability of myeloma cells to internalize and store iron, cytofluorimetric analysis of LIP formation was carried out in myeloma cells after 24 h of treatment with FAC. Our data showed a significant increase of LIP value (ΔF = 0.29 ± 0.09) in PCs treated with FAC compared to control (Fig. 1C).

**Fig. 1:**
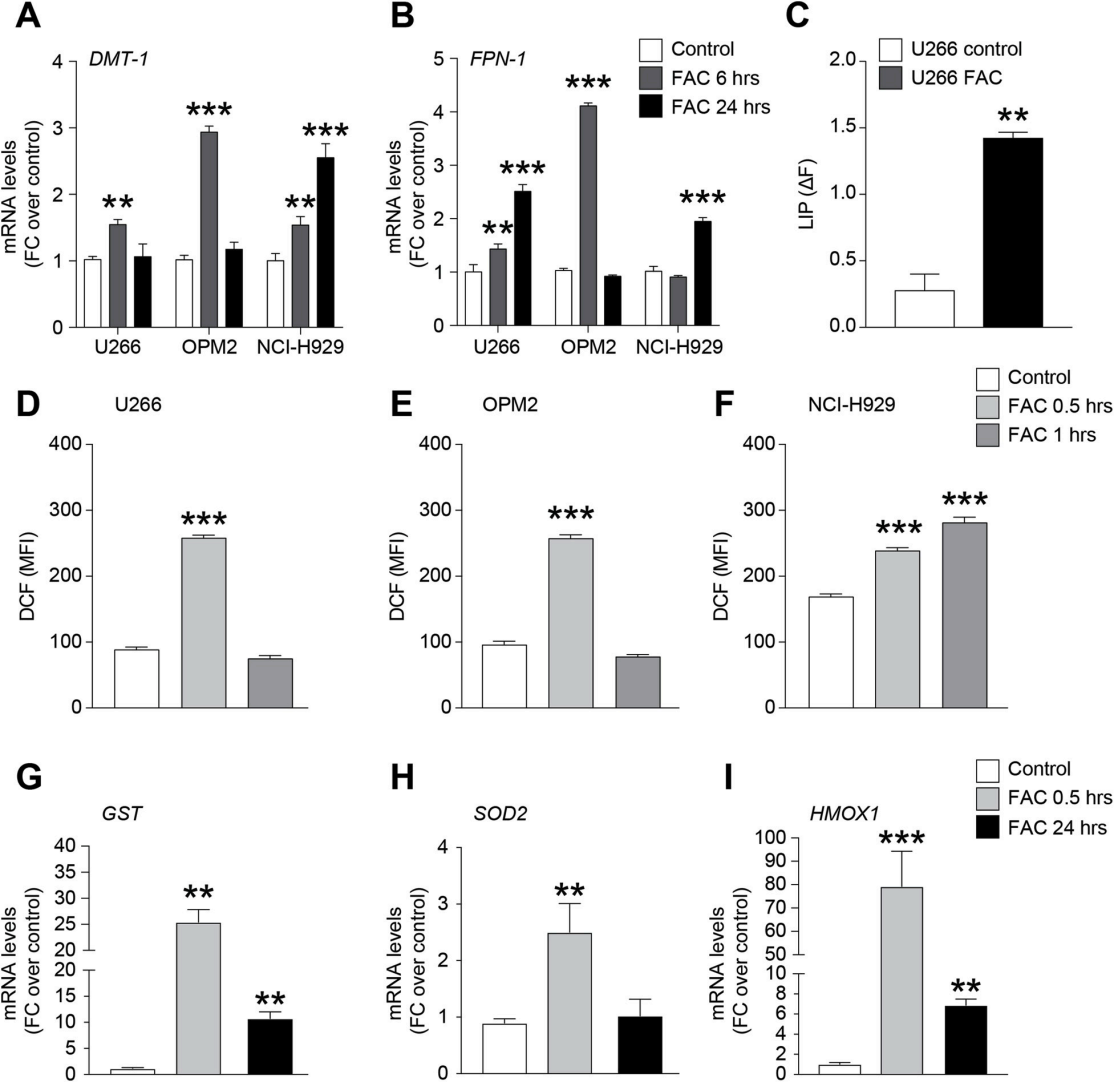
**Myeloma cell lines intake and accumulate iron.** DMT-1 (A) and FPN-1 (B) mRNA levels in U266, OPM2 and NCI–H929 myeloma cell lines at 6 and 24 h after 400 μM FAC exposure; data are expressed as mean of FC over control; C) LIP evaluated in U266 cells; data are expressed as mean of ΔF ± SEM; ΔF = MFI_CA-AM/DF_-MFI_CA-AM_. **p-value <0.01 and ***p-value <0.001 *versus* control. D-F). Cellular ROS production evaluated in U266 (D), OPM2 (E) and NCI–H929 (F) cells after 0.5 and 1 h FAC exposure; data are expressed as mean of dichlorofluorescein (DCF) fluorescence intensity (MFI) ± SEM; G-I) GST (G), SOD2 (H) and HMOX1 (I) mRNA levels in U266 cell line after 0.5 and 24 h treatment with FAC; data are expressed as mean of FC over control. **p-value <0.01 and ***p-value <0.001 *versus* control.

### 3.2 PCs respond to iron-induced oxidative stress by upregulating scavenger-related genes and increasing mitochondrial content

Since iron is involved in free radical generating reactions, we then analyzed the cellular redox status of HMCLs during exposure to FAC. ROS levels increased about 2 folds after 0.5 h exposure to FAC and returned to normal levels within 1 h from treatment (Fig. 1D–F). Only NCI–H929 still showed significative high ROS levels after 1 h compared to control. Interestingly, myeloma cells reacted to the higher ROS production inducing a concomitant activation of antioxidant-related genes such as glutathione S-transferase (GST; FC over control: 25.4 ± 1.7), superoxide dismutase 2 (SOD2; FC over control: 2.45 ± 0.2) and heme oxygenase 1 (HMOX-1; FC over control: 76.9 ± 6.5) as soon as 0.5 h after starting FAC treatment (Fig. 1G–I). Consistently, the increased levels of ROS were associated to a transient depolarization of mitochondria at 6 h post-FAC exposure (Fig. 2A). Indeed, by using flow cytometry, we observed that DiOC_2_(3) staining intensity decreased in U266 (FAC: 51.0 ± 10.5 *versus* control: 135.6 ± 6.2; MFI) and NCI–H929 cells (FAC 6 h: 96.1 ± 12.9 *versus* control: 166.53 ± 20.9; MFI) and in both cases restored after 24 h treatment (Fig. 2B). Notably, OPM2 cell line did not show significant modifications of the mitochondrial membrane polarization at both 6 h and 24 h post FAC exposure.

**Fig. 2:**
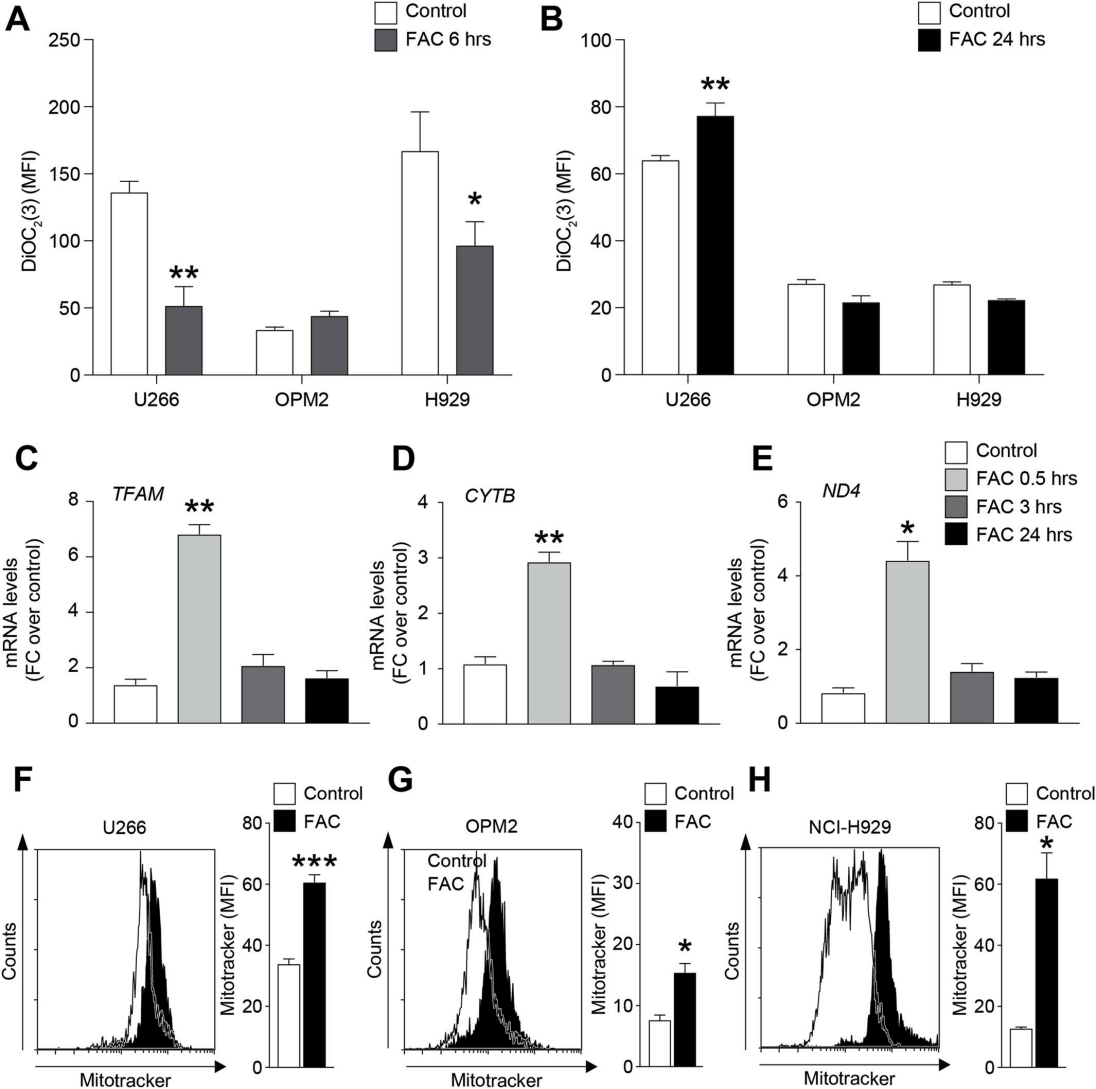
**Iron increases myeloma cell lines mitochondrial content.** A-B) Mitochondrial membrane potential evaluated in HMCLs stained with DiOC_2_(3); data are shown as mean of MFI ± SEM. C-E) TFAM (C), CYTB (D) and ND4 (E) mRNA levels in U266 cell line after 0.5, 3 and 24 h FAC exposure; data are expressed as mean of FC over control ± SEM. F–H) Representative plots and quantification of mitochondrial mass analysis using MitoTracker probe in HMCLs 24 h after FAC treatment; data are shown as mean of MFI ±SEM. *p-value < 0.05, **p-value < 0.01 and ***p-value < 0.001 *versus* control.

Interestingly, qRT-PCR analysis showed that PCs responded to FAC-induced oxidative and mitochondrial stress by upregulating the mitochondrial transcription factor A (TFAM), a gene involved in mitochondrial biogenesis and energetic metabolism (FC over control at 0.5 h: 6.8 ± 0.24; Fig. 2C). This increase was coupled with a significant upregulation of the mRNA levels of cytochrome *b* (CYTB; FC over control at 0.5 h: 2.9 ± 0.1; Fig. 2D) and NADH dehydrogenase subunit 4 (ND4; FC over control at 0.5 h: 4.4 ± 0.4; Fig. 2E), both components of the mitochondrial respiratory chain. Such evidences were confirmed analyzing the mitochondrial mass by measuring MitoTracker staining using flow cytometry. After 24 h FAC treatment, HMCLs showed higher mitochondrial mass (Fig. 2F–H). To confirm the mitochondrial functionally [44] we performed HPLC assay for quantify the energetic metabolites after FAC treatment. We observed that FAC treatment induced an increase of ATP, ATP/ADP ratio and sum of triphoshates nucleosides (Table 1). We observed an increase of energy charge potential (ECP) value after 24 h of the treatment with FAC. Moreover, iron was able to induce NAD^+^/NADH ratio in U266 cells (Table 1).

**Table 1:** Changes in the metabolic parameters related to mitochondrial function and energy metabolism in U266 cells after exposure to FAC.

	ATP	ATP/ADP	ECP	Sum of triphosphate nucleosides	NAD^+^/NADH
Control U266	4.19 ± 0.15	3.10 ± 0.12	0.83 ± 0.03	7.01 ± 0.28	10.64 ± 1.94
U266 + FAC	5.29 ± 0.52*	4.89 ± 0.66*	0.88 ± 0.02*	9.68 ± 0.84*	22.55 ± 4.67*

Values are the means ± standard deviations of five different experiments and are expressed as nmol/10^6^ cells.

ECP = Energy Charge Potential calculated as ATP +1/2ADP/ATP + ADP + AMP.

Sum of triphosphate nucleosides = ATP + GTP + UTP + CTP.

*significantly different from controls, p < 0.05.

### 3.3 Iron promotes bortezomib resistance in myeloma PCs

**Fig. 3:**
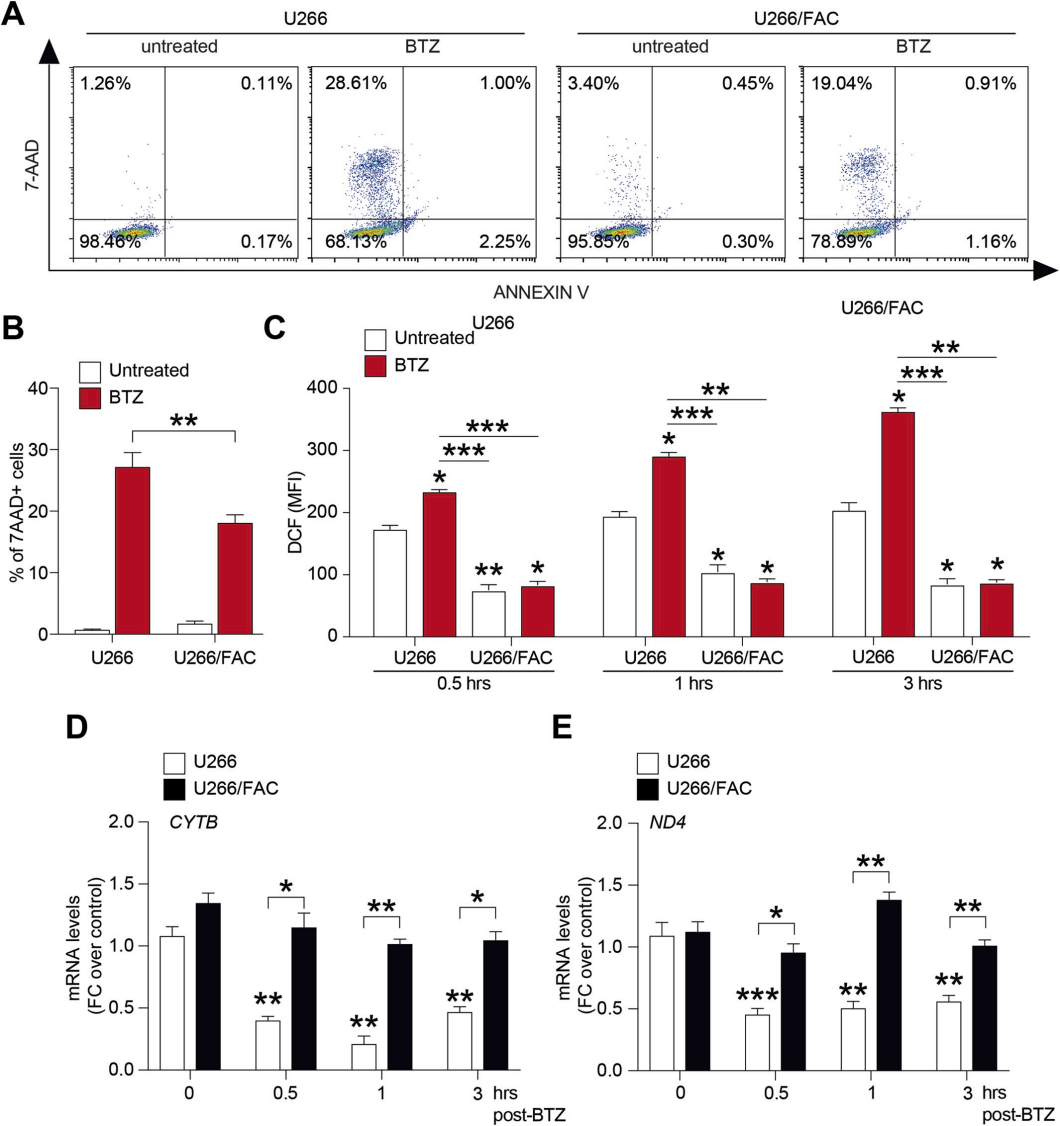
**Iron induces bortezomib resistance in myeloma PCs.** A-B) Representative dot plots (A) and quantification (B) of the percentage of apoptotic cells after 24 h treatment with15 nM BTZ on the viability in U266 and U266/FAC cells; data are expressed as mean % ± SEM; **p-value < 0.01 between groups. C) Cellular ROS production evaluated in U266 cells after 0.5, 1 and 3 h post BTZ treatment; data are expressed as dichlorofluorescein (DCF) MFI ± SEM. D) CYTB and ND4 mRNA levels in U266 cell line after 0.5, 1 and 3 h BTZ treatment; data are expressed as mean of FC over control ±SEM. E) Cellular ROS production evaluated in U266/FAC cells after 0.5, 1 and 3 h BTZ treatment; data are expressed as mean of DCF MFI ±SEM. F) CYTB and ND4 mRNA levels in U266/FAC cells after 0.5, 1 and 3 h BTZ exposure; data are expressed as mean of FC over control ±SEM. **p-value < 0.01 and ***p-value < 0.001; ^##^p-value < 0.01 and ^###^p-value < 0.001*versus control.*

To determine whether iron could affect BTZ-induced apoptosis in myeloma PCs, U266 cell line was used as iron-overloaded cell model by exposing PCs to 24 h FAC (U266/FAC cells). Following 24 h treatment with 15 nM BTZ, analysis of cell viability revealed that U266/FAC cells showed reduced percentage of apoptotic cells as compared to PCs not pretreated with FAC (U266) (U266/FAC + BTZ: 19.05 ± 0.5% *versus* U266 + BTZ: 28.75 ± 0.6%; % of apoptotic cells; Fig. 3A and B), thus supporting the hypothesis that iron mediates resistance to BTZ-induced cytotoxicity in myeloma cells. Taking into account that oxidative stress is an important mechanism of BTZ cytotoxicity, we next evaluated changes of ROS levels after BTZ exposure both in U266 and U266/FAC cells. Drug treatment caused a progressive increase of ROS levels up to 3 h in U266 cells (from 194.0 ± 14.0 to 294.9 ± 6.4 and 199.0 ± 21.0 to 357.4 ± 8.2, respectively at 1 and 3 h; MFI; Fig. 3C) while no changes of ROS levels were observed in U266/FAC cells (Fig. 3C). Consistently, we also observed a significant down-regulation of mRNA levels of CYTB (FC over control: 0.4 ± 0.05, 0.22 ± 0.1 and 0.48 ± 0.07 respectively at 0.5, 1 and 3 h, Fig. 3D) and ND4 (FC over control: 0.5 ± 0.06, 0.56 ± 0.04 and 0.6 ± 0.02 respectively at 0.5, 1 and 3 h, Fig. 3E) in U266 cells treated with the PI. In contrast, BTZ-treated U266/FAC cells did not show any significant variation in expression levels of the same energy metabolism-related genes (Fig. 3D and E).

### 3.4 U266/FAC cells showed decreased sensitivity to bortezomib *in vivo*

**Fig. 4:**
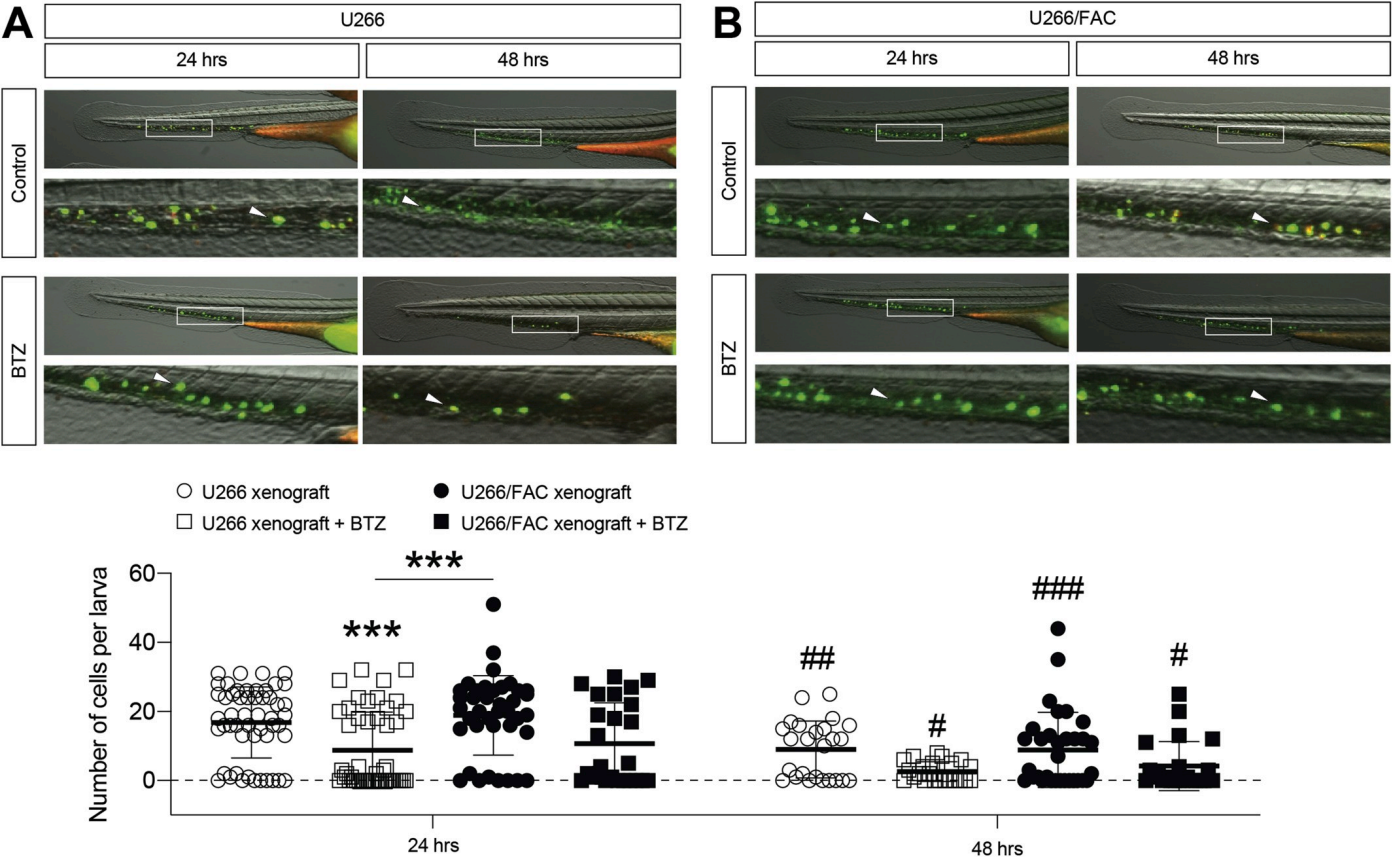
**Iron reduces bortezomib sensitivity of engrafted myeloma PCs in zebrafish larval model.** A) Representative image and stereological quantification of engrafted U266 (A) and U266/FAC (B) cells in zebrafish larvae after 24 and 48hrs BTZ treatment; data are shown as scattered dot plots and mean ± SD (each dot represents a larva); ***p-value <0.001 *versus* control, ^#^p-value<0.05, ^##^p < 0.01, ^###^p-value<0.001 *versus* corresponding 24 h, Mann-Whitney *t*-test.

In order to further confirm *in vivo* that iron is able to induce BTZ resistance in PCs, U266 and U266/FAC cells were injected in zebrafish larvae of 2 days post fertilization (dpf) and their invasiveness analyzed following BTZ treatment of larvae by bath immersion. Stereological quantification of engrafted myeloma cells in the caudal hematopoietic tissue (CHT) revealed a significant reduction of U266 cells in BTZ-treated larvae after both 24 and 48 h compared to untreated zebrafish (respectively 8.75 ± 1.6 *versus* 16.8 ± 1.4 and 9.0 ± 1.6 *versus*2.6 ± 0.6; number of engrafted U266 cells per larva) (Fig. 4A). Importantly, BTZ-treated larvae injected with U266/FAC did not show significant reduction of engrafted PCs as compared to untreated control group (Fig. 4B).

### 3.5 Iron treatment is able to promote M2-like polarization in human monocytes

**Fig. 5:**
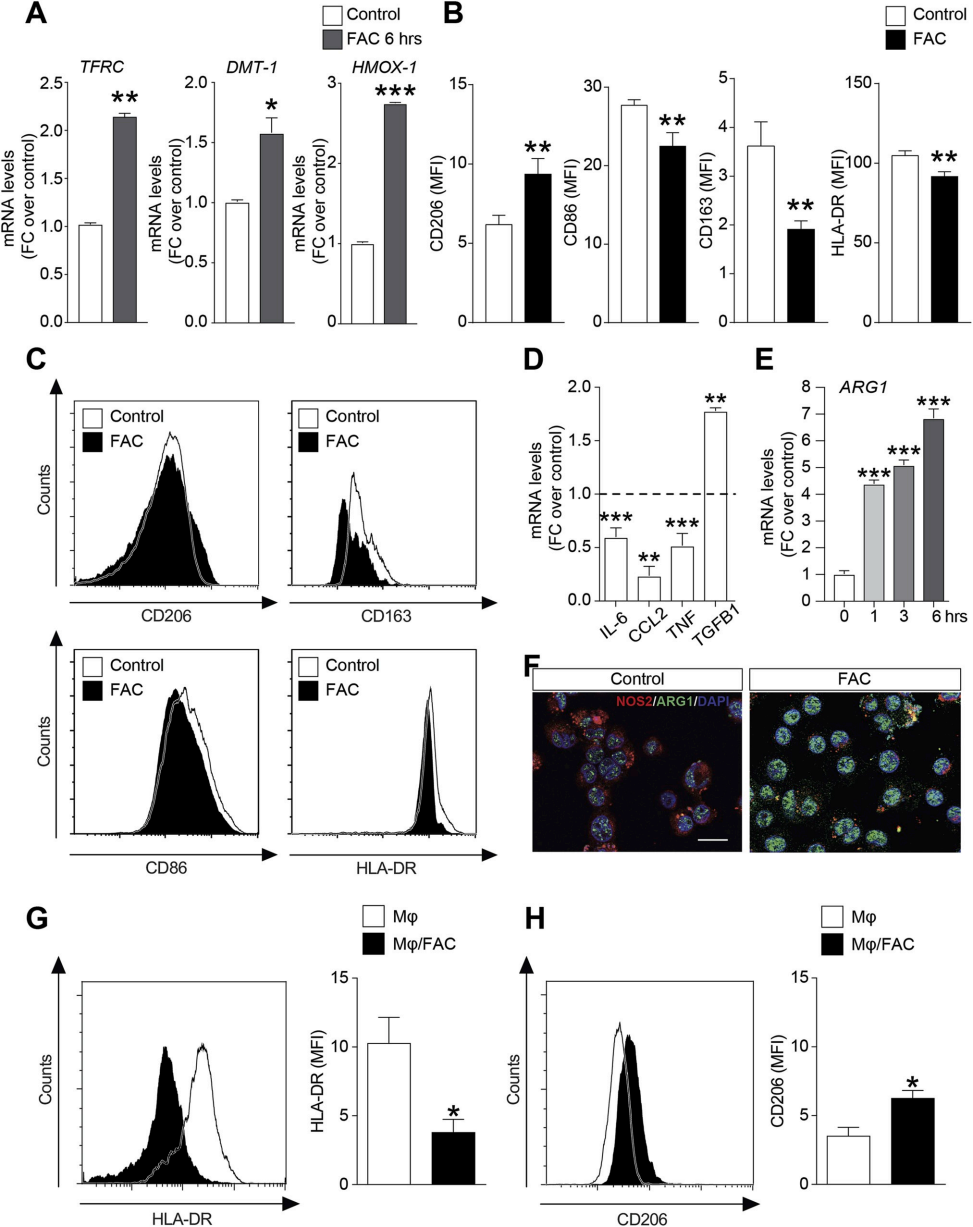
**Iron promotes immuno-suppressive phenotype in human monocytes.** (A) TFRC, DMT-1 and HMOX-1 mRNA levels in U937 cell line after 6 h 100 μM FAC treatment; data are expressed as mean FC over control ± SEM; B–C) Flow cytometric analysis of CD206, CD163, CD86 and HLA-DR in U937 cell line after FAC treatment. D) IL-6, CCL2, TNFα, TGFβ1 and mRNA levels in U937 cells after 24 h FAC exposure; data are expressed as mean of FC over control ± SEM; E) ARG1 mRNA levels after 1, 3 and 6 h FAC treatment; data are expressed as mean of FC over control ± SEM; F) Immunofluorescence pictures of ARG1 (green) and NOS2 (red) expression in control and U937 cells treated with FAC. Scale bar: 20 μm. Representative plots and quantifications of HLD-DR (H) and CD206 (G–H) MFI in human healthy donors-derived primary Mϕ after 24 h FAC exposure; data are shown as mean of MFI ± SEM. *p-value < 0.05, **p-value < 0.01 and ***p-value < 0.001 *versus* control. (For interpretation of the references to colour in this figure legend, the reader is referred to the Web version of this article.)

We also investigated the role of iron in myeloma tumor microenvironment focusing our attention on the effects of FAC exposure in human monocytes. Using U937 monocytic cell line as model, we found that FAC significantly up-regulated DMT1 and HMOX1 gene expression (Fig. 5A). Importantly, iron treatment did not affect U937 cell viability (Supplementary Fig. 2).

Subsequently, we analyzed the effect of FAC exposure on polarization balance of U937 cell lines. Compared to untreated cells, monocytes treated with FAC for 24 h increased surface expression of the classical M2 marker CD206 while decreased those of the M1 markers CD86 and HLA-DR, as evaluated by flow cytometry (Fig. 5B and C). To further confirm this evidence, mRNA levels of pro-inflammatory cytokines interleukin 6 (IL-6), C–C motif chemokine ligand 2 (CCL2) and tumor necrosis factor alfa (TNFα) and of the anti-inflammatory cytokine Transforming Growth Factor beta 1 (TGFB1) were analyzed. Consistently, we observed a reduction of IL-6 (0.6 ± 0.06, FC over control), CCL2 (0.24 ± 0.06, FC over control) and TNFα (0.52 ± 0.08, FC over control) transcript levels and a significant upregulation of those of TGFB1 (1.78 ± 0.02, FC over control) in FAC-treated monocytes (Fig. 5D). In addition, iron treatment increased the mRNA levels of arginase 1 (ARG1), a M2 marker, after 1, 3 and 6 h FAC exposure (Fig. 5F). Immunostaining further confirmed ARG1 increased protein expression and NOS2, an M1 marker, downregulation (Fig. 5F). These evidences were further confirmed on healthy donor-derived primary monocytes (Mϕ). In addition, we also observed a significant reduction of HLA-DR expression associated to increased levels of CD206 in FAC-exposed Mϕ (Fig. 5G) further pointing to the ability of FAC to promotes the M2 polarization of human macrophages.

### 3.6 Iron promotes M2 macrophage polarization and their interaction with myeloma cells *in vivo*

**Fig. 6:**
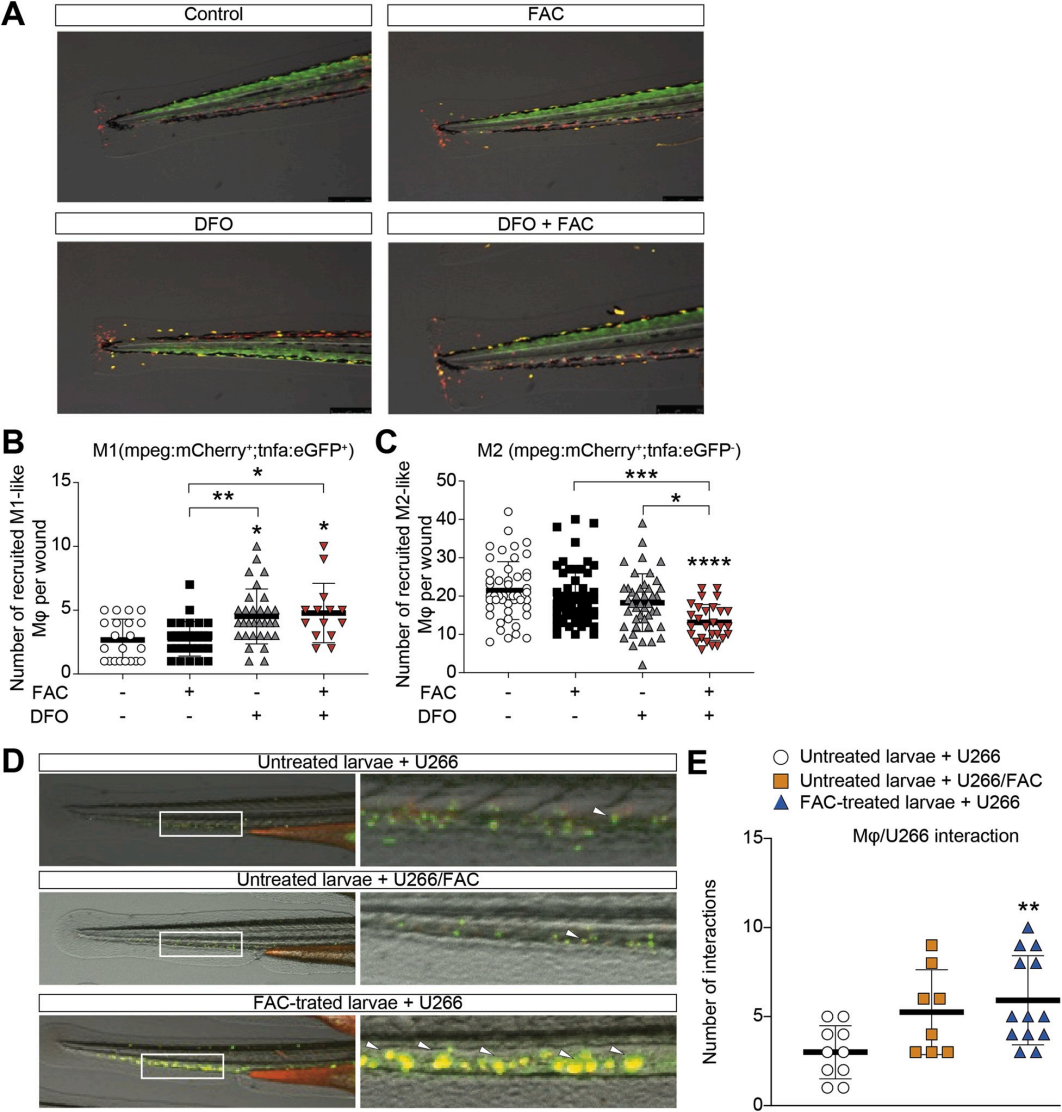
**Iron promotes M2 phenotype and PCs-macrophage interactions in zebrafish**. A) Representative images of M1 and M2 macrophages in *Tg* (*mpeg1:mCherry;tnfa: eGFP)* double transgenic larvae after 6 h treatment with FAC, DFO or their combination. B–C) Quantification of M1 (*mCherry*^+^*/eGFP*^+^: yellow) and M2 (*mCherry*^*+*^*/eGFP*^*-*^: red) macrophages in damaged area per larva. **p < 0.01 and ***p < 0,001, ANOVA and Kruskal-Wallis multiple comparisons test. D) Representative images of engrafted U266, U266/FAC cells and U266-injected larvae exposed to FAC (FAC-treated larvae) at 24 h. E) Engrafted U266 cells interacting with endogenous Mϕ per larva in the CHT 24 h post-injection. Data are shown as mean ± SD. *versus* control (larvae injected with U266 cell line); *p-value < 0.05, **p-value < 0.01 and ***p-value < 0.001 *versus* control. Each dot represents a larva. (For interpretation of the references to colour in this figure legend, the reader is referred to the Web version of this article.)

In order to fully elucidate the role of macrophages following FAC exposure, we used the *Tg(mpeg1:mCherry;tnfa:eGFP)* double transgenic larvae, in which M1 macrophages are tnfa^+^ and mpeg1^+^ double-positive, while M2 macrophages are tnfa^−^ and mpeg1^+^ [42]. Zebrafish larvae (2dpf) were treated with 100 μM FAC, 100 μM iron chelator deferoxamine (DFO) or their combination. After 24 h, caudal fin amputation was executed to trigger macrophages activation [45] and the recruitment of polarized macrophages was observed after 6 h. Data showed that FAC-treated larvae did not show increase of *tnfa* expression in recruited macrophages compared to control larvae (Fig. 6A). On the contrary, iron chelation induced by DFO treatment was able to significantly increase *tnfa* expression (about 2-fold) in recruited macrophages compared to FAC-treated and untreated larvae, thus favoring the activation of pro-inflammatory M1 phenotype (Fig. 6B). Moreover, in larvae treated with DFO/FAC a significant reduction of M2 macrophage polarization was also observed compared to FAC-treated larvae (Fig. 6C).

In order to fully dissect *in vivo* the role of iron in improving myeloma cell interaction with endogenous Mϕ favoring PC survival, we analyzed U266 and U266/FAC cells xenograft in *mfap4:tomato* transgenic zebrafish larvae. In this model, *mfap4-*driven fluorescent protein tomato (red) permits the visualization of membrane projections in migrating macrophages allowing the evaluation of their interactions with PCs (green). We observed that the larvae treated with FAC after xenotransplantation showed a significant increase of interaction between myeloma cells and endogenous Mϕ in the CHT 24 h post-engraftment respect to untreated larvae (Fig. 6D and E).

### 3.7 FAC pre-treated monocytes favour bortezomib resistance of myeloma PCs

**Fig. 7:**
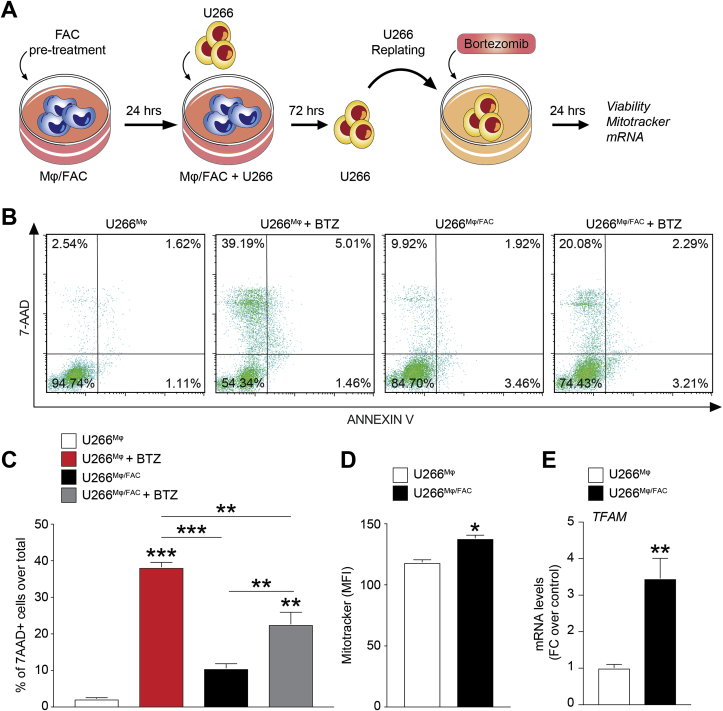
**Iron-exposed monocytes reduce bortezomib sensitivity of myeloma PCs.** A) Experimental paradigm of primary human monocytes (Mϕ) and U266 cells cocultures. Representative dot plots (B) and quantification (C) of apoptotic cells after BTZ treatment in U266 cells obtained from coculture with Mϕ (U266^Mϕ^) or from coculture with Mϕ/FAC (U266^Mϕ/FAC^). Data are shown as mean percentage ±SEM; ***p-value < 0.001 and **p-value < 0.01 versus controls or between groups. D) Quantification of mitochondrial mass analysis using MitoTracker probe in U266^Mϕ^ and U266^Mϕ/FAC^ cells; data are shown as mean of MFI ±SEM; E) TFAM mRNA levels U266^Mϕ^ and U266^Mϕ/FAC^ cells; data are expressed as mean of FC over control ±SEM; *p-value < 0.05 and **p-value < 0.01 *versus* control.

As it has been demonstrated that tumor associated macrophages (TAMs) promote cancer resistance through releasing iron in tumor macroenvironment, we subsequently investigated the ability of monocytes pre-treated for 24 h with FAC (Mϕ/FAC cells) to release iron, thus favouring BTZ resistance of myeloma PCs. U266 cells were co-cultured with Mϕ/FAC cells or not pre-treated monocytes (Mϕ) for 72 h and then collected, re-plated and treated with 15 nM BTZ for 24 h (Fig. 7A). As shown in Fig. 7B–C, U266 cells isolated from the co-culture with Mϕ/FAC exhibited 12% apoptotic cells compared to 36% of PCs co-cultured with Mϕ. Interestingly, we also observed increased mitochondrial mass in U266 isolated from co-culture with Mϕ/FAC compared with untreated Mϕ (138.4 ± 1.6 *versus* 118.6 ± 1.3; MFI; Fig. 7D) confirmed by increased expression of TFAM (FC over controls: 3.5 ± 0.4; Fig. 7E).

## 4 Discussion

Despite the continuous progress made in recent decades, the pathogenesis of MM remains to be fully elucidated. One of the issues to be addressed is a detailed analysis of the role played by high concentration of intracellular iron in PCs, and the effects resulting from its dysregulation [27]. The current study demonstrates that excess iron favours myeloma resistance to BTZ both after a direct uptake of iron by PCs and through iron-releasing monocytes. Iron trafficking in mammalian cells is controlled by complex mechanisms including both uptake from serum transferrin through the endocytic pathway by TFR and non-transferrin-bound iron DMT1 and iron export by FPN1. Herein we observed that exposure to FAC induces iron trafficking in HMCLs by upregulating DMT-1 and FPN1 genes, showing the ability of FAC-exposed MM cell lines to increase respectively the iron uptake and extrusion in order to maintain intracellular iron homeostasis. Iron is known to be involved in intracellular ROS-generating reactions which can change the cellular redox status [46]. As expected, FAC exposure triggered ROS production in HMCLs, associated to a subsequent upregulation of scavenger-related genes (GST, SOD and HMOX1), a transient depolarization of mitochondria, upregulation of the genes involved in the mitochondrial respiratory chain (CYTB and ND4) and mitochondrial biogenesis (TFAM), with an increase of mitochondrial mass. These data suggest that FAC treatment induces ROS levels sufficient to stimulate a cellular adaptive response in myeloma PCs that avoid apoptosis through the activation of the cellular scavenging system and the enhancement of mitochondrial mass and activity. Therefore, our data further confirm previous evidences showing that cellular LIP is not a simply cellular source of pro-oxidant iron but it may also serve as a target of oxidative and nitrosative species [47]. These results are consistent with previous observations showing that FAC induced Nrf2 pathway activation which may be responsible for upregulation of genes involved in chemoresistant phenotype [48]. To this regard, we have also previously showed that Nrf2 activation occurs following BTZ treatment in myeloma cells conferring chemoresistance via the HO-1 pathway [49]. Taken all together these observations along with the presented data might suggest that iron supplementation confers resistance via a direct effect of the Nrf2 axis. In addition, cellular iron content has been demonstrated to influence mitochondrial biogenesis [50]. TFR, which is expressed at increased levels in rapidly proliferating cells and malignant ones, including myeloma PCs, has also been reported to be involved in regulating mitochondrial function, supporting mitochondrial respiration [51]. Depletion of cellular iron by using DFO decreases the expression of mitochondrial metabolism-associated genes in murine myoblast cells; on the contrary, up-regulation of PGC1α expression decreases the expression of FPN1 and induces up-regulation of TFR [50]. Consistently, MM patients who have a high mitochondrial metabolism also display low levels of FPN1 linked to a poor prognosis [50].

We have recently shown that BTZ-resistant MM cells show better mitochondrial fitness and more efficient oxidative respiration (OXPHOS) compared to the sensitive counterparts. In accordance with these findings, we studied whether iron-induced mitochondrial biogenesis could affect BTZ-induced apoptosis. U266/FAC cells showed reduced cytotoxic effects after BTZ treatment compared to BTZ-treated U266, demonstrating that iron is involved in myeloma PI-resistance. A mechanism of BTZ-induced cell death is ROS-driven caspase-dependent apoptosis [52]. Thus, to understand why the iron decreases BTZ efficacy, we first analyzed the dynamics of ROS in U266 and U266/FAC cells. BTZ treatment was able to increase ROS levels in U266 cell line followed by the downregulation of energy-metabolism related genes. On the contrary in U266/FAC cells, the amount of ROS remained almost constant and no significant changes in CYTB and ND4 gene expression were observed. To confirm whether iron can affect BTZ sensitivity *in vivo,* zebrafish larvae were injected with U266 or U266/FAC cells and subsequently treated with the PI. After injection, MM cells can home or metastasize to the BM-like niche region, the CHT [53]. The quantification of engrafted PCs in the CHT revealed a significant reduction of the number of U266 cells in BTZ-treated larvae compared to untreated animals, while iron pre-treated MM cells did not show significant changes of the total number of engrafted PCs. BTZ selectively induces apoptosis in myeloma PCs by inducing components of the proapoptotic/terminal unfolded protein response (UPR) and triggering endoplasmic reticulum (ER) stress [49]. The UPR is a signalling activated by the accumulation of unfolded protein within ER, which attempts to reduce the protein load on the ER and increase its folding capacity. However, unresolved ER stress results in the activation of apoptosis with the transition of the UPR from a protective to an apoptotic response. There is increasing evidence for a complex interplay between the UPR and iron homeostasis: ER stress can modify the expression of iron-related genes and iron excess is associated with increased expression of ER chaperones [[54], [55], [56]], thus increasing ER stress response. These findings support our data demonstrating that iron excess promotes BTZ resistance.

Bone marrow microenvironment plays a pivotal role in supporting tumor cell growth, disease progression and drug resistance of myeloma PCs. Macrophages are key components of the myeloid infiltrate in tumors [57], including MM, where these myeloid cells have been reported to negatively impact disease course [25]. It has also been demonstrated that patients with active MM have a higher number of macrophages in the BM microenvironment, which support PCs proliferation and favours chemotherapy-induced apoptosis [58]. MM cells specifically recruit monocytes secreting chemo-attractive factors such as CXCL12 and blockage with anti-CXCR4 antibodies significantly abrogates monocyte recruitment [25]. As macrophages display different phenotype and function depending on environmental signals, it is conceivable that once recruited into the MM microenvironment, monocytes acquire an alternative M2 polarization promoting tumor growth, enhancing angiogenesis and immunosuppression [59]. A physiological function of macrophages is to maintain the iron balance and an increase of iron traffic by TAM can promote tumor progression and chemotherapy resistance [24]. Apoptotic tumor cells trigger macrophages towards an iron releasing phenotype [23] and combination of chemotherapy with iron chelator therapy could reduce drug resistance [60,61].

We showed that FAC-treatment promotes M2-like monocytes with CD206^high^CD86^low^HLA-DR^low^ phenotype together with a downregulation of pro-inflammatory cytokines (IL-6, CCL2, TNFα mRNA) and increased expression of anti-inflammatory markers (ARG1 and TGFB1), thus suggesting the establishment of an iron-mediated immune suppressive tumor microenvironment favoring MM cells growth and survival. These results were furtherly supported *in vivo* using *Tg(mpeg1:mCherry;tnfa:eGFP)* double transgenic larvae, in which FAC treatment did not induce the activation of pro-inflammatory M1-like monocyte phenotype after inflammation triggered by fin amputation. Iron chelation induced by DFO treatment was able to revert the effect of FAC increasing *tnfa* expression in recruited macrophages and also reduced the total number of M2-like macrophages.

In addition to the direct effect of iron on macrophage polarization, our results also indicate that FAC treatment increases the ability of myeloma PCs to recruit macrophages. Indeed, *mfap4:tomato* transgenic zebrafish larvae injected with MM cells and exposed to FAC showed a significant increase of interactions between PCs and endogenous Mϕ compared to untreated larvae. Moreover, FAC-exposed xenografted larvae showed a significantly higher number of invading PCs, suggesting a potential role of iron in favoring tumor metastasis and migration. Importantly, physical interactions between macrophages and MM cells have been shown to protect PCs from drug-induced apoptosis [62]. Therefore, as TAM can release iron, we finally co-cultured myeloma cells with monocytes pre-treated or not with FAC. As expected, U266 cells isolated from co-culture with Mϕ/FAC exhibited reduced BTZ-induced apoptosis compared with MM cells obtained from co-culture with untreated Mϕ, suggesting an iron release from FAC pre-treated monocytes to myeloma PCs.

In summary, our results demonstrated that iron has profound impacts both on PCs and tumor microenvironment. Indeed, iron trafficking, by modifying energetic metabolism of cancer cells and impairing inflammatory status of macrophages, represents a critical regulator of myeloma cell-macrophage interactions, shaping the MM tumor niche and promoting BTZ-resistance. Further, it could be shown that iron chelation treatment in combination with BTZ improves the PI-cytotoxic effects and/or could overcome drug resistance.

## Declaration of competing interest

The authors declare that they have no competing interests.

## Figures and Tables

**figs1:**
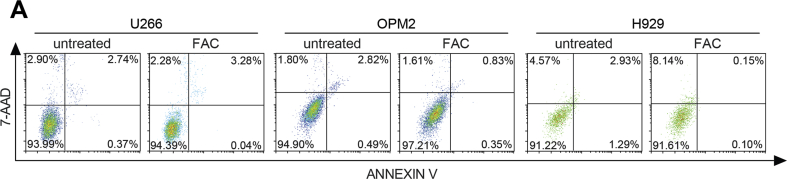


**figs2:**
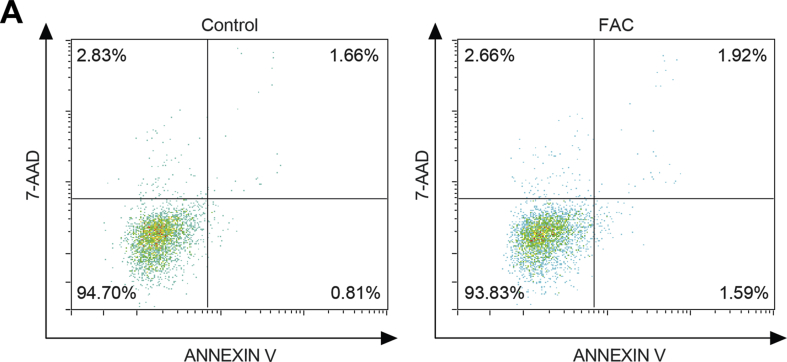


## References

[1] Dimopoulos M.A., Richardson P.G., Moreau P., Anderson K.C. (2015). Current treatment landscape for relapsed and/or refractory multiple myeloma. Nat. Rev. Clin. Oncol..

[2] Jemal A., Siegel R., Ward E., Murray T., Xu J., Thun M.J. (2007). Cancer statistics. Ca - Cancer J. Clin..

[3] Ria R., Vacca A. (2020). Bone marrow stromal cells-induced drug resistance in multiple myeloma. Int. J. Mol. Sci..

[4] Anderson C.P., Shen M., Eisenstein R.S., Leibold E.A. (2012). Mammalian iron metabolism and its control by iron regulatory proteins. Biochim. Biophys. Acta.

[5] Dixon S.J., Stockwell B.R. (2014). The role of iron and reactive oxygen species in cell death. Nat. Chem. Biol..

[6] Thevenod F. (2018). Iron and its role in cancer defense: a double-edged sword. Met. Ions Life Sci..

[7] Manz D.H., Blanchette N.L., Paul B.T., Torti F.M., Torti S.V. (2016). Iron and cancer: recent insights. Ann. N. Y. Acad. Sci..

[8] Brookes M.J., Hughes S., Turner F.E., Reynolds G., Sharma N., Ismail T., Berx G., McKie A.T., Hotchin N., Anderson G.J., Iqbal T., Tselepis C. (2006). Modulation of iron transport proteins in human colorectal carcinogenesis. Gut.

[9] Trinder D., Zak O., Aisen P. (1996). Transferrin receptor-independent uptake of differic transferrin by human hepatoma cells with antisense inhibition of receptor expression. Hepatology.

[10] Richardson D.R., Baker E. (1990). The uptake of iron and transferrin by the human malignant melanoma cell. Biochim. Biophys. Acta.

[11] Parenti R., Salvatorelli L., Magro G. (2014). Anaplastic thyroid carcinoma: current treatments and potential new therapeutic options with emphasis on TfR1/CD71. Internet J. Endocrinol..

[12] Campisi A., Bonfanti R., Raciti G., Bonaventura G., Legnani L., Magro G., Pennisi M., Russo G., Chiacchio M.A., Pappalardo F., Parenti R. (2020). Gene silencing of transferrin-1 receptor as a potential therapeutic target for human follicular and anaplastic thyroid cancer. Mol. Ther. Oncolytics.

[13] Benadiba J., Rosilio C., Nebout M., Heimeroth V., Neffati Z., Popa A., Mary D., Griessinger E., Imbert V., Sirvent N., Peyron J.F. (2017). Iron chelation: an adjuvant therapy to target metabolism, growth and survival of murine PTEN-deficient T lymphoma and human T lymphoblastic leukemia/lymphoma. Leuk. Lymphoma.

[14] Hagag A.A., Badraia I.M., Abdelmageed M.M., Hablas N.M., Hazzaa S.M.E., Nosair N.A. (2018). Prognostic value of transferrin receptor-1 (CD71) expression in acute lymphoblastic leukemia. Endocr. Metab. Immune Disord. - Drug Targets.

[15] Callens C., Coulon S., Naudin J., Radford-Weiss I., Boissel N., Raffoux E., Wang P.H., Agarwal S., Tamouza H., Paubelle E., Asnafi V., Ribeil J.A., Dessen P., Canioni D., Chandesris O., Rubio M.T., Beaumont C., Benhamou M., Dombret H., Macintyre E., Monteiro R.C., Moura I.C., Hermine O. (2010). Targeting iron homeostasis induces cellular differentiation and synergizes with differentiating agents in acute myeloid leukemia. J. Exp. Med..

[16] VanderWall K., Daniels-Wells T.R., Penichet M., Lichtenstein A. (2013). Iron in multiple myeloma. Crit. Rev. Oncog..

[17] Whitnall M., Howard J., Ponka P., Richardson D.R. (2006). A class of iron chelators with a wide spectrum of potent antitumor activity that overcomes resistance to chemotherapeutics. Proc. Natl. Acad. Sci. U. S. A..

[18] Campanella A., Santambrogio P., Fontana F., Frenquelli M., Cenci S., Marcatti M., Sitia R., Tonon G., Camaschella C. (2013). Iron increases the susceptibility of multiple myeloma cells to bortezomib. Haematologica.

[19] Gu Z., Wang H., Xia J., Yang Y., Jin Z., Xu H., Shi J., De Domenico I., Tricot G., Zhan F. (2015). Decreased ferroportin promotes myeloma cell growth and osteoclast differentiation. Canc. Res..

[20] Sharma S., Nemeth E., Chen Y.H., Goodnough J., Huston A., Roodman G.D., Ganz T., Lichtenstein A. (2008). Involvement of hepcidin in the anemia of multiple myeloma. Clin. Canc. Res..

[21] Katodritou E., Ganz T., Terpos E., Verrou E., Olbina G., Gastari V., Hadjiaggelidou C., Varthaliti M., Georgiadou S., Westerman M., Zervas K. (2009). Sequential evaluation of serum hepcidin in anemic myeloma patients: study of correlations with myeloma treatment, disease variables, and anemia response. Am. J. Hematol..

[22] Knutson M.D., Oukka M., Koss L.M., Aydemir F., Wessling-Resnick M. (2005). Iron release from macrophages after erythrophagocytosis is up-regulated by ferroportin 1 overexpression and down-regulated by hepcidin. Proc. Natl. Acad. Sci. U. S. A..

[23] Jung M., Weigert A., Mertens C., Rehwald C., Brune B. (2017). Iron handling in tumor-associated macrophages-is there a new role for lipocalin-2?. Front. Immunol..

[24] Prill S., Rebstock J., Tennemann A., Korfer J., Sonnichsen R., Thieme R., Gockel I., Lyros O., Monecke A., Wittekind C., Weimann A., Grosser K., Wiechmann V., Kubick C., Bechmann I., Lordick F., Kallendrusch S. (2019). Tumor-associated macrophages and individual chemo-susceptibility are influenced by iron chelation in human slice cultures of gastric cancer. Oncotarget.

[25] Beider K., Bitner H., Leiba M., Gutwein O., Koren-Michowitz M., Ostrovsky O., Abraham M., Wald H., Galun E., Peled A., Nagler A. (2014). Multiple myeloma cells recruit tumor-supportive macrophages through the CXCR4/CXCL12 axis and promote their polarization toward the M2 phenotype. Oncotarget.

[26] Bordini J., Galvan S., Ponzoni M., Bertilaccio M.T., Chesi M., Bergsagel P.L., Camaschella C., Campanella A. (2017). Induction of iron excess restricts malignant plasma cells expansion and potentiates bortezomib effect in models of multiple myeloma. Leukemia.

[27] Kamihara Y., Takada K., Sato T., Kawano Y., Murase K., Arihara Y., Kikuchi S., Hayasaka N., Usami M., Iyama S., Miyanishi K., Sato Y., Kobune M., Kato J. (2016). The iron chelator deferasirox induces apoptosis by targeting oncogenic Pyk2/beta-catenin signaling in human multiple myeloma. Oncotarget.

[28] Tibullo D., Di Rosa M., Giallongo C., La Cava P., Parrinello N.L., Romano A., Conticello C., Brundo M.V., Saccone S., Malaguarnera L., Di Raimondo F. (2015). Bortezomib modulates CHIT1 and YKL40 in monocyte-derived osteoclast and in myeloma cells. Front. Pharmacol..

[29] Hideshima T., Ikeda H., Chauhan D., Okawa Y., Raje N., Podar K., Mitsiades C., Munshi N.C., Richardson P.G., Carrasco R.D., Anderson K.C. (2009). Bortezomib induces canonical nuclear factor-kappaB activation in multiple myeloma cells. Blood.

[30] Zhao S., Zhang L., Xu Z., Chen W. (2013). Neurotoxic effects of iron overload under high glucose concentration. Neural Regen. Res..

[31] Agoro R., Taleb M., Quesniaux V.F.J., Mura C. (2018). Cell iron status influences macrophage polarization. PloS One.

[32] Guo C., Chen X., Xiong P. (2014). Baicalin suppresses iron accumulation after substantia nigra injury: relationship between iron concentration and transferrin expression. Neural Regen. Res..

[33] Vicario N., Pasquinucci L., Spitale F.M., Chiechio S., Turnaturi R., Caraci F., Tibullo D., Avola R., Gulino R., Parenti R., Parenti C. (2019). Simultaneous activation of mu and delta opioid receptors reduces allodynia and astrocytic connexin 43 in an animal model of neuropathic pain. Mol. Neurobiol..

[34] Barresi V., Romano A., Musso N., Capizzi C., Consoli C., Martelli M.P., Palumbo G., Di Raimondo F., Condorelli D.F. (2010). Broad copy neutral-loss of heterozygosity regions and rare recurring copy number abnormalities in normal karyotype-acute myeloid leukemia genomes. Genes Chromosomes Cancer.

[35] Tibullo D., Barbagallo I., Giallongo C., La Cava P., Branca A., Conticello C., Stagno F., Chiarenza A., Palumbo G.A., Di Raimondo F. (2012). Effects of second-generation tyrosine kinase inhibitors towards osteogenic differentiation of human mesenchymal cells of healthy donors. Hematol. Oncol..

[36] Vicario N., Bernstock J.D., Spitale F.M., Giallongo C., Giunta M.A.S., Li Volti G., Gulisano M., Leanza G., Tibullo D., Parenti R., Gulino R. (2019). Clobetasol modulates adult neural stem cell growth via canonical hedgehog pathway activation. Int. J. Mol. Sci..

[37] Gulino R., Vicario N., Giunta M.A.S., Spoto G., Calabrese G., Vecchio M., Gulisano M., Leanza G., Parenti R. (2019). Neuromuscular plasticity in a mouse neurotoxic model of spinal motoneuronal loss. Int. J. Mol. Sci..

[38] Tibullo D., Longo A., Vicario N., Romano A., Barbato A., Di Rosa M., Barbagallo I., Anfuso C.D., Lupo G., Gulino R., Parenti R., Li Volti G.L., Palumbo G.A., Di Raimondo F.D., Giallongo C. (2020). Ixazomib improves bone remodeling and counteracts sonic hedgehog signaling inhibition mediated by myeloma cells. Cancers.

[39] Whitlock K.E., Westerfield M. (2000). The olfactory placodes of the zebrafish form by convergence of cellular fields at the edge of the neural plate. Development.

[40] Walton E.M., Cronan M.R., Beerman R.W., Tobin D.M. (2015). The macrophage-specific promoter mfap4 allows live, long-term analysis of macrophage behavior during mycobacterial infection in zebrafish. PloS One.

[41] White R.M., Sessa A., Burke C., Bowman T., LeBlanc J., Ceol C., Bourque C., Dovey M., Goessling W., Burns C.E., Zon L.I. (2008). Transparent adult zebrafish as a tool for in vivo transplantation analysis. Cell Stem Cell.

[42] Nguyen-Chi M., Laplace-Builhe B., Travnickova J., Luz-Crawford P., Tejedor G., Phan Q.T., Duroux-Richard I., Levraud J.P., Kissa K., Lutfalla G., Jorgensen C., Djouad F. (2015). Identification of polarized macrophage subsets in zebrafish. Elife.

[43] de Oliveira S., Reyes-Aldasoro C.C., Candel S., Renshaw S.A., Mulero V., Calado A. (2013). Cxcl8 (IL-8) mediates neutrophil recruitment and behavior in the zebrafish inflammatory response. J. Immunol..

[44] Tibullo D., Giallongo C., Romano A., Vicario N., Barbato A., Puglisi F., Parenti R., Amorini A.M., Wissam Saab M., Tavazzi B., Mangione R., Brundo M.V., Lazzarino G., Palumbo G.A., Volti G.L., Raimondo F.D., Lazzarino G. (2020). Mitochondrial functions, energy metabolism and protein glycosylation are interconnected processes mediating resistance to bortezomib in multiple myeloma cells. Biomolecules.

[45] Nguyen-Chi M., Laplace-Builhe B., Travnickova J., Luz-Crawford P., Tejedor G., Lutfalla G., Kissa K., Jorgensen C., Djouad F. (2017). TNF signaling and macrophages govern fin regeneration in zebrafish larvae. Cell Death Dis..

[46] Daghman N.A., Elder G.E., Savage G.A., Winter P.C., Maxwell A.P., Lappin T.R. (1999). Erythropoietin production: evidence for multiple oxygen sensing pathways. Ann. Hematol..

[47] Damasceno F.C., Condeles A.L., Lopes A.K.B., Facci R.R., Linares E., Truzzi D.R., Augusto O., Toledo J.C. (2018). The labile iron pool attenuates peroxynitrite-dependent damage and can no longer be considered solely a pro-oxidative cellular iron source. J. Biol. Chem..

[48] Silva-Gomes S., Santos A.G., Caldas C., Silva C.M., Neves J.V., Lopes J., Carneiro F., Rodrigues P.N., Duarte T.L. (2014). Transcription factor NRF2 protects mice against dietary iron-induced liver injury by preventing hepatocytic cell death. J. Hepatol..

[49] Tibullo D., Barbagallo I., Giallongo C., Vanella L., Conticello C., Romano A., Saccone S., Godos J., Di Raimondo F., Li Volti G. (2016). Heme oxygenase-1 nuclear translocation regulates bortezomibinduced cytotoxicity and mediates genomic instability in myeloma cells. Oncotarget.

[50] Zhan X., Yu W., Franqui-Machin R., Bates M.L., Nadiminti K., Cao H., Amendt B.A., Jethava Y., Frech I., Zhan F., Tricot G. (2017). Alteration of mitochondrial biogenesis promotes disease progression in multiple myeloma. Oncotarget.

[51] Senyilmaz D., Virtue S., Xu X., Tan C.Y., Griffin J.L., Miller A.K., Vidal-Puig A., Teleman A.A. (2015). Regulation of mitochondrial morphology and function by stearoylation of TFR1. Nature.

[52] Weniger M.A., Rizzatti E.G., Perez-Galan P., Liu D., Wang Q., Munson P.J., Raghavachari N., White T., Tweito M.M., Dunleavy K., Ye Y., Wilson W.H., Wiestner A. (2011). Treatment-induced oxidative stress and cellular antioxidant capacity determine response to bortezomib in mantle cell lymphoma. Clin. Canc. Res..

[53] Sacco A., Roccaro A.M., Ma D., Shi J., Mishima Y., Moschetta M., Chiarini M., Munshi N., Handin R.I., Ghobrial I.M. (2016). Cancer cell dissemination and homing to the bone marrow in a zebrafish model. Canc. Res..

[54] You K.R., Liu M.J., Han X.J., Lee Z.W., Kim D.G. (2003). Transcriptional regulation of the human transferrin gene by GADD153 in hepatoma cells. Hepatology.

[55] Vecchi C., Montosi G., Zhang K., Lamberti I., Duncan S.A., Kaufman R.J., Pietrangelo A. (2009). ER stress controls iron metabolism through induction of hepcidin. Science.

[56] Haney S.L., Varney M.L., Safranek H.R., Chhonker Y.S., GD N., Talmon G., Murry D.J., Wiemer A.J., Wright D.L., Holstein S.A. (2019). Tropolone-induced effects on the unfolded protein response pathway and apoptosis in multiple myeloma cells are dependent on iron. Leuk. Res..

[57] Kawano Y., Moschetta M., Manier S., Glavey S., Gorgun G.T., Roccaro A.M., Anderson K.C., Ghobrial I.M. (2015). Targeting the bone marrow microenvironment in multiple myeloma. Immunol. Rev..

[58] Berardi S., Ria R., Reale A., De Luisi A., Catacchio I., Moschetta M., Vacca A. (2013). Multiple myeloma macrophages: pivotal players in the tumor microenvironment. J. Oncol..

[59] Vacca A., Ribatti D. (2006). Bone marrow angiogenesis in multiple myeloma. Leukemia.

[60] Ninomiya T., Ohara T., Noma K., Katsura Y., Katsube R., Kashima H., Kato T., Tomono Y., Tazawa H., Kagawa S., Shirakawa Y., Kimura F., Chen L., Kasai T., Seno M., Matsukawa A., Fujiwara T. (2017). Iron depletion is a novel therapeutic strategy to target cancer stem cells. Oncotarget.

[61] Miyamoto T., Kashima H., Yamada Y., Kobara H., Asaka R., Ando H., Higuchi S., Ida K., Mvunta D.H., Shiozawa T. (2016). Lipocalin 2 enhances migration and resistance against cisplatin in endometrial carcinoma cells. PloS One.

[62] Zheng Y., Cai Z., Wang S., Zhang X., Qian J., Hong S., Li H., Wang M., Yang J., Yi Q. (2009). Macrophages are an abundant component of myeloma microenvironment and protect myeloma cells from chemotherapy drug-induced apoptosis. Blood.

