# Supplementary material for: Iron regulates myeloma cell/macrophage interaction and drives resistance to bortezomib
Source: Redox Biol. 2020 Jun 24;36:101611. doi: 10.1016/j.redox.2020.101611 (PMC7327252; doi:10.1016/j.redox.2020.101611)
Supplement: Multimedia component 1 [file mmc1.docx]

**Supplementary figures**

**
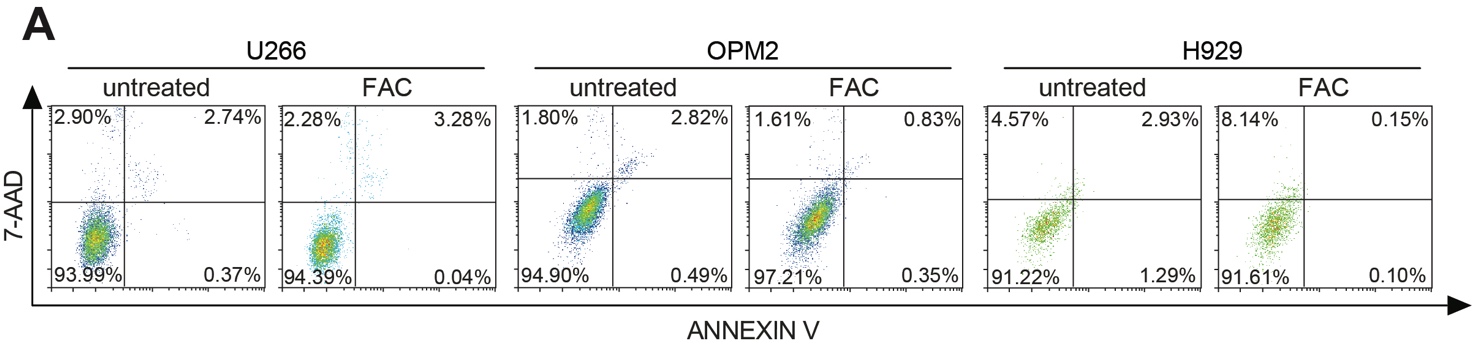
Figure S1.** A) Representative density plot of 7-AAD and ANNEXIN V cytofluorimetric analysis on U266, OPM2 and NCI-H929 myeloma cell lines untreated controls and cultures exposed to 400 μM FAC for 24 hrs; data represent the percentage of gated cells.

**
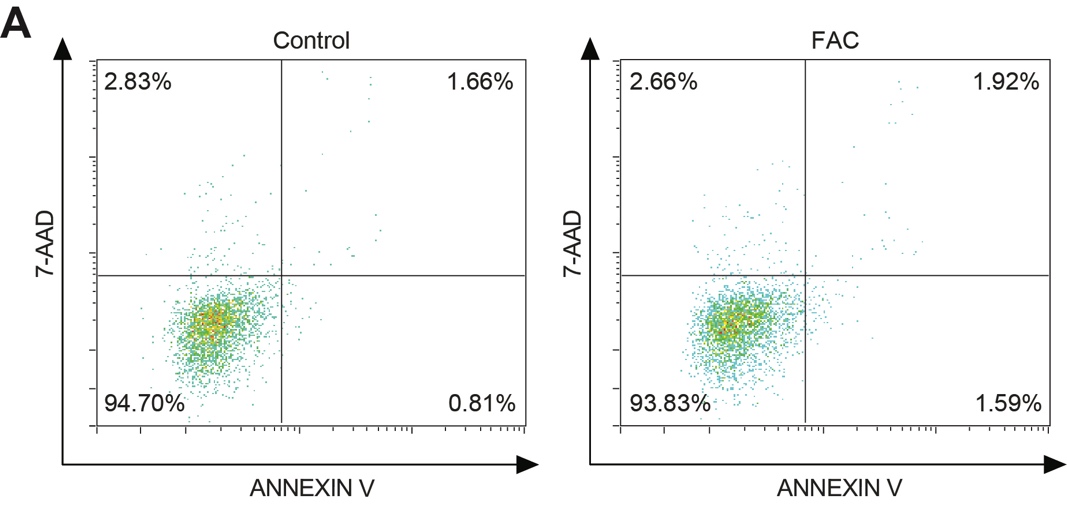
**

**Figure S2.** A) Representative dot plots of annexin-V-FITC and 7-ADD staining in U937 cells exposed to 100 μM FAC for 24 hrs; data represent the percentage of gated cells.

**Supplementary methods**

**Materials**

**Flow cytometry**

To evaluate apoptosis after drug treatment, cells were stained with annexin AV FITC/7-ADD assay kit (Beckman Coulter, Mylan, Italy) according to the manufacturer’s instruction. After centrifugation, cells were resuspended in PBS, stained with annexin AV FITC/7-ADD assay kit. The apoptotic population was immediately evaluated by flow cytometry.
